# Complex Systems in Phase Space

**DOI:** 10.3390/e22101103

**Published:** 2020-09-29

**Authors:** David K. Ferry, Mihail Nedjalkov, Josef Weinbub, Mauro Ballicchia, Ian Welland, Siegfried Selberherr

**Affiliations:** 1 School of Electrical, Computer, and Energy Engineering, Arizona State University, Tempe, AZ 25287-5706, USA; iwelland@asu.edu; 2 Institute for Microelectronics, TU Wien, 1040 Vienna, Austria; mihail.nedialkov@tuwien.ac.at (M.N.); mauro.ballicchia@tuwien.ac.at (M.B.); siegfried.selberherr@tuwien.ac.at (S.S.); 3 Bulgarian Academy of Sciences, 1113 Sofia, Bulgaria; 4 Christian Doppler Laboratory for High Performance TCAD, Institute for Microelectronics, TU Wien, 1040 Vienna, Austria; josef.weinbub@tuwien.ac.at

**Keywords:** nonlinearity, hysteresis, quantum transport, non-Hermitian behavior

## Abstract

The continued reduction of semiconductor device feature sizes towards the single-digit nanometer regime involves a variety of quantum effects. Modeling quantum effects in phase space in terms of the Wigner transport equation has evolved to be a very effective approach to describe such scaled down complex systems, accounting from full quantum processes to dissipation dominated transport regimes including transients. Here, we discuss the challanges, myths, and opportunities that arise in the study of these complex systems, and particularly the advantages of using phase space notions. The development of particle-based techniques for solving the transport equation and obtaining the Wigner function has led to efficient simulation approaches that couple well to the corresponding classical dynamics. One particular advantage is the ability to clearly illuminate the entanglement that can arise in the quantum system, thus allowing the direct observation of many quantum phenomena.

## 1. Introduction

Over the past few decades, technological momentum has pushed semiconductors to the nanometer scale and has even led to structural modifications of the basic field-effect transistor (FET), such as replacing the planar FET with the finFET or Trigate FET using a vertical channel [1,2]. In such devices, the active region is restricted to nanometer-scale dimensions in one or more directions, which, depending on the involved time and energy scales, gives rise to quantum effects, such as energy quantization, tunneling, position-momentum uncertainty, phase coherence, etc., and these make the carrier transport quite complex. The complexity arises not merely from the need for a deeper understanding of transport and behavior of such small systems, but also because these small devices interact strongly with their environment. From a physical point of view, a small device comprises an active region, which is open to the environment in which it is embedded (and thus exchanges carriers is subject to interactions with this environment). This connection to the environment may be through a set of portals, described as contacts, or through interactions with the phonon structure of the lattice upon which the device lies, or through other types of interactions. The central feature of such small devices is that the device micro-dynamics cannot be treated in isolation and must be considered in conjunction with this environment [3]. This leads to a central tenet that the transport is now heavily influenced by this coupling to the environment, and the basic Liouville equation and its causal boundary conditions must be modified to account for the influence of this environment. In particular, the environment dramatically changes the quantum nature of the device, and the device similarly must have an effect upon the environment, as measurements can only be made in the environment [4]. In considering transport in these small systems, further complexity arises because it has lost its time reversible properties. A phase transition to irreversible behavior has occurred [5]. Hence, the device in particular is now a far-from-equilibrium, complex system.

There is an additional problem in small semiconductor devices, which further complicates the behavior. An analysis of the classical transport always gives us insight into the challenges that arise in quantum transport models. In small devices, the time scale of carrier transport, within the device, may well be dominated by the transient response characteristics of the carrier velocity and distribution function [6,7]. Then, there may be excitation/relaxation effects in the environment, which affect the transient behavior of the device itself through the device-environment interactions. Each of these effects provides considerable complications and sets requirements upon any approach to quantum transport to be applied in such nano-devices. This is not the least because the transient excitation response is usually quite different from the relaxation response. As may be expected, this further complicates the far-from-equilibrium treatment of the device.

When we drive a semiconductor system out of equilibrium, the resulting distribution function used to characterize the transport does not simply evolve from the equilibrium version. Rather, it evolves into a balance between the driving forces and the dissipative forces (assuming that a steady-state balance can be achieved). There is a hierarchy of equations used to determine this distribution function, as we move from large classical systems to smaller fully quantum mechanical systems, and, in fact, there is a hierarchy of quantum mechanical approaches. The most detailed classical approach uses the Boltzmann transport equation, and this transitions to several “Boltzmann-like” quantum analogs, which arise from the density matrix, the Wigner function and non-equilibrium Green’s functions as one moves down the hierarchy. Much has been written about quantum transport, especially with regard to semiconductor devices. Unfortunately, a great deal of this material has not taken proper account of the far-from-equilibrium behavior that these devices exhibit, the difficulties of short-time response, or the complicated interactions between the environment and the device. 

Our purpose in this article is not to review this entire body of work, but rather to try to illustrate the nature of the problems that face someone trying to make sense of the complex system with which one desires to work. In the next section, we will discuss some of the attributes of complex systems, whether classical or quantum, as well as how classical and quantum systems differ. In Section 3, we will explore the difficulties that arise due to the arrow of time and the resulting irreversibility. Then, in the following sections, we discuss the leading methods of quantum transport and their advantages and disadvantages. This will mention the nonequilibrium Green’s functions but mainly rely upon phase-space Wigner functions. We will actually do these last two in the reverse order, because we believe the former is limited in complex systems, while the latter allows us to utilize numerical tools—the ensemble Monte Carlo particle methods—that are directly transferred from classical transport. We will illustrate the methods with new results from four different applications, some of which have never been studied with Monte Carlo methods previously.

## 2. The Nature of Complex Quantum Systems

A complex system may be regarded to be any system that is composed of multiple parts, many of which are interacting with one another, which fits the above description of the device and its environment perfectly. This assembly of various parts are typically both nonlinear and inhomogeneous, especially when we also consider the environment in which the system is embedded. For our purposes, we can thus regard any electronic nano-device as being a complex system, as this device certainly interacts with its environment in a way that changes the properties of both the device and the environment, a point we deal with below in detail. 

To begin, let us consider the system itself without its environment. We know that the set of energy levels for a quantum object allow us to express the Hamiltonian in terms of the extracted eigen-states for these levels; this is the energy eigen-state description and the Hamiltonian is diagonal in the absence of interactions, as

(1) $$H_{0}\varphi_{n}\left(x,t\right)=E_{0n}\varphi_{n}\left(x,t\right),$$

where the $\left\{\varphi_{n}\left(x,t\right)\right\}$ form a complete set of basis functions as mentioned. For interactions within the system, such as with scattering as an interaction, we treat the time-dependent perturbation with the Fermi-golden rule. But, this does not conserve normalization of the state. To conserve normalization, we have to use self-consistency, which in this case means accounting for the decay of the initial state [8]. When this is done, one determines a self-energy given as $\Sigma_{n}=\Delta_{n}+i\Gamma_{n}$. The real part of the self-energy corresponds to a shift downward of the energy (frequency shift due to the energy shift $E_{0n}\to E_{0n}-\Sigma_{n}$). The imaginary part of the self-energy provides a damping of the state in time, which is the analog of the classical resistive damping of a resonant circuit. The diagonal energies in the Hamiltonian have this self-energy correction for each energy level (as indicated, Σ may be different for each energy level), and the Hamiltonian has become non-Hermitian. In particular, an arrow of time has entered the description of our quantum object. More importantly, the interaction leads to the view that states which do not lie on the energy shell (described by the classical delta function between energy and its evaluation in terms of momentum) can be important in the transport. So, there is no longer any single energy shell, but a range of momentum values that can have the same energy. To describe this, one defines a new quantity, which is called the spectral density, and describes the relationship between the energy and the momentum, typically a Lorentzian line in equilibrium systems, just as in the classical case. We will deal with the spectral density further below.

Let us now embed our quantum object, the device, within a surrounding environment as discussed above. We can describe the states of the device by a density matrix *ρ_D_* and the environment by a density matrix *ρ_E_*, which form a tensor product when the two parts are uncoupled. Our goal is to see how they occur after the coupling and the interaction. The complex system can now be defined by a Hamiltonian containing three terms:

(2) $$H=H_{D}+H_{E}+H_{int}$$

where *H_D_* describes the device, *H_E_* describes the environment, and *H_int_* describes interactions between the two. The composite density matrix for the entire system begins with the tensor product of the two density matrices described above. There are two crucial steps in defining a reduced density matrix for just the desired parts of the device. The first is to project out these states via a projection super-operator. The second is to perform a trace over the environmental states which yields just the reduced set of pointer states. This procedure has been known for a considerable time [9,10,11], and the derivations of the following form have been discussed extensively [3,12,13]. The result is the transport equation for the projected/desired part of the device

(3) $$i\hbar \frac{\partial \rho_{PD}}{\partial t}=\left(H_{PD}+H\prime_{int}\right)\rho_{PD}+\Sigma \rho_{PD}$$

where

(4) $$\begin{array}{c} H\prime_{int}\rho_{PD}=Tr_{E}\left\{PH_{int}P\rho_{D}\right\}\;\;\;\;\;\\ \Sigma \rho_{PD}=Tr_{E}\left\{C\rho_{PD}\right\}\;\;\;\;\;\;\;\;\;\\ \;\;\;\;\;\;\;\;C=\frac{i}{\hbar }PHQe^{-iQHQt/\hbar }QHP \end{array},$$

and *P* and *Q* = 1 − *P* are the projection super-operators that project the desired dynamics of the device onto a reduced density matrix, or to its conjugate parts, respectively. Once again, the net Hamiltonian is non-Hermitian. Moreover, the response of (3) is retarded, as is the usual case in projected systems. More importantly, the second term in the parentheses of (3) may contain new processes that are not part of either the device or the environment alone. Writing the second term in parentheses as separated, as in (3), is a short-hand notation, since *ρ_D_* cannot actually be separated from this interaction term. In that sense, this term is an entanglement between environment and device, except that “entanglement” is also not a good description as this connection can appear even in classical systems (entanglement is usually reserved for quantum systems). This term can be new processes depending upon both the environment and the system. One such type is the resonant back-scattering trajectories from quantum dots in a magnetic field [14,15,16], which depends critically upon the actual confinement structure of the device and its contacts. 

However, how does *ρ_D_* differ from *ρ_PD_* = *Pρ_D_*? The former density matrix contains all the eigen-states of the entire device, given in (1), while the latter contains only those which can be used e.g., for modeling and simulation of the entire system. The separation arises from the coupling to the environment, which may well wash out a number of the quantum states. Those states which are not washed out, termed the pointer states, provide the quantum effects within the device. In a bulk semiconductor, this might just be the carrier dynamics of spatially quantized electrons (or holes). For the device, it at least contains all eigen-states which are necessary to describe the response of the device to excitations. For a counter example, the device contains the oxide, but the oxide polar modes are usually not seen in the response directly, yet they provide intra-device scattering processes as does the interface between the oxide and the semiconductor. These scattering processes are included above in *C*, as are those processes arising from environmental effects, such as lattice phonons. The form of *C* is standard perturbation theory where *PHQ* and *QHP* are versions of the matrix element coupling the device to the phonons and its adjoint, respectively, while the exponential contributes to the energy conservation through the appropriate frequencies. We now find that quantum transport, like classical transport has distinctly far-from-equilibrium behavior which can lead to non-Hermitian Hamiltonians and very nonlinear, inhomogeneous, and retarded transport, with new phenomena that are not present in equilibrium systems.

### 2.1. Environmentally-Induced States

Classically, a well known environmentally-induced effect in the device world is drain-induced barrier lowering [17]. Current injection into the MOS channel is governed by a potential barrier at the source end of the channel. The barrier height is controlled by the gate-source potential difference. Normally, there is enough scattering in the channel of the injected carriers, that the drain voltage is “screened” from this barrier. But, if ballistic transport begins to occur as the size of the transistor is reduced, the screening effect of the scattering is also significantly reduced. Then, the potential barrier begins to be affected by the drain potential which, in turn, can act to lower the barrier itself, letting more current into the channel [18]. We consider this as an environmentally-induced state as the drain voltage is an environmental variable whose effect is transmitted through one of the contacts of the device structure. The contacts themselves are a complex object whose properties are often not well behaved. Hence, the device does not actually see the real drain voltage, but only some complex image of it as transmitted into the actual device.

Now, let’s turn to a quantum interaction. We pointed out above that the coupling between the environment and the device can lead to new states which do not exist in the device alone. We can illustrate this with an array of quantum dots which are open to each other and to the environment. Any two dots are coupled to each other via an open (non-tunneling) point contact-like structure, and the end dots of the array are coupled similarly to the environment. It has been found that such a structure supports new states that are localized on the quantum point contacts [14]. These states are stable states and contribute strongly to the overall conductance through the device, and have been shown to support the concept of quantum Darwinism [19], in which the exact same wave function amplitude is seen in each mode that propagates through the quantum point contact.

### 2.2. Trajectories and Quantum Mechanics

Trajectories have been used in classical mechanics for centuries, where a particle trajectory follows Hamilton’s equations of motion. And, this has been extended to transport theory through a particle based simulation of the Boltzmann transport equation, which we normally call a kinetic Monte Carlo process [20]. But, shortly after the appearance of the Schrödinger equation [21], trajectories were suggested for quantum mechanics as well. Physical observables retain the same appearance as their classical counterparts, following the contributions of Madelung [22] and Kennard [23], who pointed out the quantum dynamics would follow the classical potential plus any quantum potential.

In several simulations, we have compared classical trajectories with the full quantum mechanical solutions [24,25,26]. In ballistic cases, the pointer states in classical simulations are located around singularities called centers, as the eigenstate sits on a closed ring located at this point. The full quantum simulation is, of course, smoothed out, due to uncertainty. Nevertheless, a Husimi function (which is a smoothed Wigner function) for the quantum solution is located over the center corresponding to the classical orbit. In the case of the environmentally-induced state, we term this classical state as a bipartite state that is associated with a ring of attractors located in the quantum point contact region, and the quantum state projects onto this same phase-space region. Most of these simulations actually use an iterated form of the Schrödinger equation to obtain the quantum wave functions.

Over the past few decades, it has been realized that the Wigner function is particularly suited to simulation with an ensemble of particles through the Monte Carlo procedure [27,28,29]. This is because of the strong connection between the Wigner transport equation and the Boltzmann transport equation, discussed below. A particle model to be evaluated with a Monte Carlo technique has been associated with the Wigner and the Wigner–Boltzmann equation. This model makes the analogy between classical and Wigner transport formalisms even closer, but is certainly in keeping with the approach of Kennard. We will develop this more fully in Section 4 below.

## 3. Time Irreversibility

It was pointed out above that, when we apply fields or forces to the device, the entire complex system undergoes a phase transition that breaks time-reversal symmetry. The device, within its environment, then seeks a steady-state, far-from-equilibrium stable state that balances the driving forces and the dissipative forces. During the transient response toward this stable state, if it exists, the system may evolve though a number of intermediate phases, e.g., homogeneous, inhomogeneous, linear, nonlinear, etc. When the forces are removed, the system response does not reverse its course through these different phases, but seeks a relaxation toward the equilibrium steady-state from which it initially deviated. The excitation process generates entropy [30]. The relaxation does not remove this entropy from the system but generates even more as dissipation still occurs. We briefly will describe such a case and then consider another, more complicated example. 

**Figure 1:**
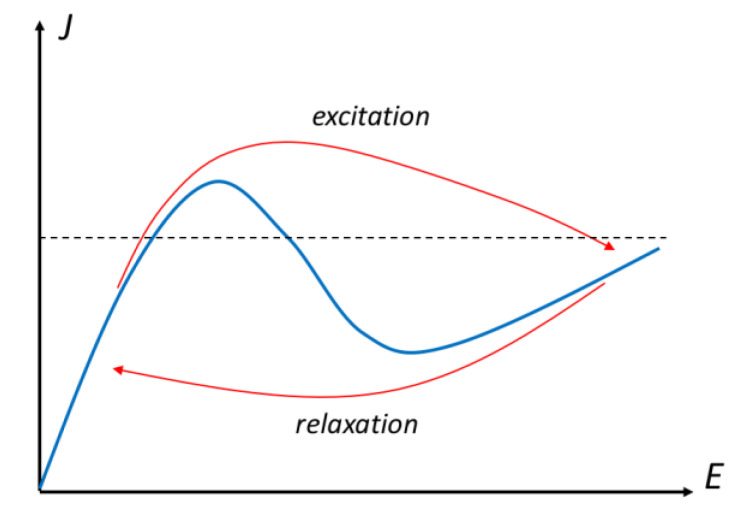
The current-field curve for an NDC device (blue curve). The red arrows indicate the differences between excitation and relaxation, explained further in the text.

To illustrate the difference between excitation and relaxation, consider the so-called Gunn diode, composed of a bulk GaAs device. Typically, it is moderately doped in the 10^14^–10^16^ cm^−3^ region. This device is an example of a negative-differential conductance (NDC) device [31]. The typical current-voltage curve is illustrated in Figure 1. Here, there are regions of the curve where a single current density can be supported by multiple values of electric field (see the dashed line). Hence, we think of this curve as *J*(*E*), and thus of conductance. Thus, the use of NDC. It is important to note that, for the dashed line, the central crossing of the *J*(*E*) curve, where *J* is decreasing while *E* is increasing, is unstable and certainly is not a position of steady state.

When the device of Figure 1 is excited, the current increases along the blue curve until the peak is reached in the current. Then, the operating point will try to jump to the high electric field region. But the path it takes will not follow the blue curve, but one closer to the red curve marked “excitation”. It may even move to a much higher current if the switching is fast, as any overshoot will appear as a higher current. So, the exact red path followed depends upon how fast the device is driven to a high-electric field. If the device reaches a steady-state, perhaps with a current between the peak and the valley, an inhomogeneous electric field and charge density will exist in the device (the horizontal dashed line in Figure 1 represents an average current for the inhomogeneous electric field). There will be a high-electric-field region and a low-electric-field region, and an inhomogeneous charge density is required to support this field difference through Gauss’ law. Now, it may turn out that it is not possible to reach a steady state, for the reason that the carriers are moving with the drift velocity appropriate for the dashed curve. As the carriers reach the drain and exit the device, the field will try to become homogeneous once again and this will trigger another high-field region near the cathode. This then propagates through the device until it reaches the anode and starts the process over again. This leads to current (and voltage) oscillations first observed by Gunn [32]. When the voltage excitation is removed, the operating point does not pass back over the peak current density, but follows the lower red arrow marked “relaxation” through the valley current density, then jumping back to the low-field current. This leads to a hysteresis in the current-voltage characteristics. When the device and its environment are considered, the nonlinear hysteretic behavior is considered to be an elegant example of catastrophe theory [33], in which the effective potential generates a so-called fold catastrophe.

We conclude that carrier transport in complex quantum systems, such as nano-scale semiconductor devices embedded within a complicated environment, requires a complex quantum description, which takes into account quantum-coherent phenomena, as well as dissipative processes of scattering, with both modified by the interaction with the environment. In the following, we will discuss the physically intuitive Wigner phase-space function [34,35,36]. Our preferred focus is upon this function, mainly because of its adaptability to ensemble Monte Carlo (EMC) simulations, and we will finally describe a number of examples of this approach.

## 4. Wigner Functions

In general, we seek a formulation of many-body statistical quantum mechanics using the methods of the interaction representation, perturbation theory, and second quantization in terms of expectation values of field operators. To begin, we need to clarify just what this last sentence means in practice. In Section 2, we broadly introduced the density matrix. The usefulness of this lies in the existence of an entire set of basis functions, which are characteristic of the problem at hand. These lead to a density matrix as

(5) $$\rho \left(x,x^{\prime },t\right)=\sum_{nm}c_{nm}\varphi_{m}^{†}\left(x^{\prime },t\right)\varphi_{n}\left(x,t\right).$$

Normally, the trace of the density matrix, which is the magnitude squared of the basis functions in the absence of interactions, gives the average of a physical quantity as

(6) 〈A〉=Tr{Aρ}.

Temporal evolution of the density matrix is governed by the Liouville equation

(7) $$i\hbar \frac{\partial \rho }{\partial t}=\left[-\frac{\hbar^{2}}{2m}\left(\frac{\partial^{2}}{\partial x^{2}}-\frac{\partial^{2}}{\partial {x^{\prime }}^{2}}\right)+V\left(x\right)-V\left(x\prime \right)\right]\rho .$$

From this equation, one can develop an effective Boltzmann-like equation of motion, as is done below. As with the wave function approach above, the density matrix is easy to couple to the self-consistent Poisson equation for devices.

The Wigner function formalism presents a physically intuitive formulation of the quantum mechanical theory, which retains most of the classical concepts and notions. The Wigner function, which is defined in the phase space, is the quantum mechanical analog of the classical distribution function. It is a real-valued function, often called a quasi-distribution, since physical averages are obtained by classical, distribution function-based expressions. However, it allows for negative values as a result of the uncertainty relation and quantum information/entanglement. The development of the Wigner phase-space formulation of quantum mechanics, and the appropriate mathematics for operators in this formulation, has been established historically [37]. This was followed by important contributions from the work of Groenewold [38] and Moyal [39]. Importantly, the Wigner function formalism has been established as an equivalent, autonomous alternative to operator mechanics; for a recent review see [35]. A self-contained formulation of the quantum theory in terms of phase-space functions has been attained by rules for filtering the admissible quantum states from the c-number functions of the phase space. Operators in this phase-space formulation are related by novel non-commutative algebraic rules given by Moyal. Important questions, like what discriminates classical from quantum mechanical behavior in the phase space have been addressed. Indeed, in equilibrium, the Wigner function is positive definite, which essentially couples it to the Boltzmann distribution function. However, the onset of quantum effects leads to both negative excursions and non-Gaussian distribution functions. Furthermore, it has been shown that phase-space quantum mechanics recovers the operator mechanics, so that it is clear that there is a logical equivalence between the two theories [40]. 

We introduce the Wigner function in the historical manner, beginning with the mixed state, single-time density matrix (5) and the Liouville equation governing it’s evolution (7). Then, we introduce the center-of-mass coordinates for position,

(8) $$\begin{array}{cc} R=\frac{1}{2}\left(x+x\prime \right)\;, & s=x-x^{\prime }. \end{array}$$

and construct the Fourier–Weyl transform in the difference variable as

(9) $$f_{W}\left(R,p,t\right)=\frac{1}{2\pi \hbar }\overset{\infty }{\underset{-\infty }{\int }}\rho \left(R+\frac{s}{2},R-\frac{s}{2}\right)e^{-isp/\hbar }.$$

This leads to the Wigner transport equation as

(10) $$\frac{\partial f_{W}}{\partial t}+\frac{p}{m}\frac{\partial f_{W}}{\partial R}=\frac{1}{2\pi \hbar }\int dp\prime V_{W}\left(R,p-p\prime \right)f_{W}\left(R,p\prime ,t\right),$$

where the Wigner potential is given by

(11) $$V_{W}\left(R,p\right)=\int ds\;sin\left(\frac{p’s}{\hbar }\right)\left[V\left(R+\frac{s}{2}\right)-V\left(R-\frac{s}{2}\right)\right].$$

The Wigner function is integrable with respect to position or momentum, and these integrals give rise to momentum or spatial carrier density distributions, respectively. The Wigner transport Equation (10) reveals an important analogy with the classical Boltzmann equation. The left hand side represents the Liouville free-streaming operator. We recognize that the Wigner function is a quasi-distribution, but it is sufficient to determine transport coefficients, given (10). That is, the Wigner function plays the same role as the Maxwell–Boltzmann distribution classically; once the Wigner function is known, all transport properties are determined from appropriate integrals over the distribution.

If the potential *V* in (11) is a linear or quadratic function, the right-hand side reduces to the classical force acceleration term so that (10) reduces to the ballistic Boltzmann equation. If the potential is a slowly varying function in the region where *fw* is sizable (characterized by a quantity called coherence length), the latter approaches the classical distribution function. Thus, the first important property is that the Wigner formalism ensures a seamless transition from quantum mechanical to classical transport regimes. This also reveals the basic difference between classical and quantum mechanical evolution in the phase space: The former (classical evolution) is governed by the normal force, corresponding to the first derivative of the Wigner potential, while all potential derivatives are involved in the latter (quantum evolution). Indeed, Wigner himself proposed that the leading term in the quantum correction would be given by the second derivative of the total potential.

Furthermore, a scattering operator, analogous to the one used with the classical Boltzmann equation, acting on *fw* may be added to the right-hand side of (10), giving rise to the Wigner-Boltzmann equation [41]. This equation describes the electron evolution as a competition between the scales of the involved physical quantities, e.g., the competition between the accelerating forces and the dissipative forces. If one develops dimensionless parameters, corresponding to the relative strength of the energy scales of the device, the potential, the phonon energy, and the electron–phonon coupling factor, these are tied into a recently derived notion called the scaling theorem [42]. This theorem reveals a second mechanism causing a seamless transition from quantum mechanical to classical transport. Scattering causes a coherence length reduction and electron localization. An increase in the electron-phonon coupling factor shrinks the spatial interval where the electronic state remains unperturbed or ballistic. This gradually transforms, on a microscopic level, the Wigner-Boltzmann equation into the classical Boltzmann counterpart. The second important property of the Wigner function formalism is that quantum-coherent and scattering-dominated transport regimes are treated on equal footing.

### 4.1. Particle Approaches 

We already noted above that there is a view of quantum mechanics that specifically incorporates particle trajectories. Madelung took careful note of Schrödinger’s work, and immediately noted that the probability density had all the appearances of a fluid flow. Kennard quickly learned of the new developments in quantum mechanics as well, and found the quantum mechanics of a system of particles came directly from the Schrödinger equation. Moreover, he found that the particles would follow normal Hamiltonian dynamics, although the potential would have to be modified through the addition of a quantum potential. One form of this potential is often called the Bohm potential following his resurrection of the Madelung–Kennard hydrodynamic ideas [43]. Further, of course, the quantum potential in which we are interested is the Wigner potential (11). Particles naturally move in phase space, so the Wigner phase-space representation of quantum mechanics is a natural venue for Monte Carlo particle dynamics. The key problem is just how to handle the complicated integral of the Wigner potential that appears in the Wigner transport Equation (10). There have been many methods developed, but we focus here on the pseudo-particle approaches that are used in Monte Carlo.

The weighted Monte Carlo approach was formally introduced in 1992 [44]. The paths considered have the problem that there are regions in phase space in which the distribution function has little weight, or is negative. This means that the particles in the Monte Carlo simulation have low probabilities of reaching these areas and the consequent solutions are very noisy in these phase space areas. In some sense, this approach arises from older ideas of variations in importance sampling to reduce variances in Monte Carlo. The weighted Monte Carlo procedure has been shown to be especially useful in the backward Monte Carlo, where one uses the series expansion to guide a propagation path from the final state back to the initial state. The path integral is expanded in a Neumann series as above [45,46]. The use of this series then corresponds to a set of paths that contain a different number of scattering interaction events. Since the paths involve a number of scattering events, each scattering event introduces a phase proportional to

(12) $$2cos\left[q\left(x_{1}-x_{2}\right)-\omega_{q}\left(t_{1}-t_{2}\right)\right],$$

where *q* and *ω_q_* are the wave number and radian frequency of the phonon. Hence, because the scattering is nonlocal, it goes beyond the Fermi golden rule used in Boltzmann transport. Presumably, after many scattering events, multiple paths can be summed to provide the negative regions of the Wigner function. Now, we note that there are two positions and two times required to formulate the scattering process, and this is a complication for the method. Moreover, here the negative regions arise solely from the scattering processes.

The affinity method is another approach where an affinity, which may be negative, is assigned to each particle. This immediately solves the problem discussed in the last paragraph, since a negative part of the Wigner function is clearly represented by particles whose affinity is also negative. The Monte Carlo approach is set up so that two systems are solved simultaneously. The first system is the particle system, which resembles a standard classical EMC. The second system is the wave properties of the particles, the affinity. That is, all particles in the system are treated classically as whole particles, but the method accounts for fully coherent transport, but has been further generalized to account for scattering. They are scattered using normal EMC scattering techniques, and are drifted and accelerated using the standard field term deriving from the solution of the Poisson equation in the presence of the real potential, such as the tunneling barriers. However, the discontinuities in the potential are handled as boundary conditions on the solutions of the Poisson equation. That is, the potential jump is introduced in matching solutions from different regions of the solution space for the Poisson equation. Once the above operations have completed, the Wigner distribution function is calculated from the particle’s position and affinity according to [47,48,49]

(13) $$f_{W}\left(x,p\right)\sim \underset{i}{\sum }\alpha_{i}\delta \left(x-x_{i}\right)\delta \left(p-p_{i}\right),$$

where *p*_i_, *x_i_*, and *α*_i_ are the momentum, position and affinity, respectively, and the sum *i* runs over the set of particles used in the simulation. There are two points here. By using this in the Wigner equation of motion, we see that one needs to update both the classical properties (position, momentum, etc.) and the quantum properties (affinity). While the classical properties are updated by the classical forces, the affinity is updated by the quantum, or Wigner, potential. Because the collisions are classical, they have to be modified for the two times that occur in (12). Actual collision durations tend to be a few femtosecond [50]. Once we have a distribution for these times, then the nonlocality of the scattering can be easily introduced for each scattering event [51].

Another approach is the signed particle approach, in which the action of the Wigner potential gives rise to generation and annihilation processes: Any particle, with sign *a* = ± 1, and momentum *p*, generates two new secondary particles, with signs ±*a* and momenta (*p* ± *p*_1_). Both the generation rate and the distribution of momentum offsets *p*_1_ are determined by the Wigner potential. Any of the three particles can again generate new, secondary pairs, etc. An important property of the model is that two particles with opposite sign, located in the same phase space point at the same time, annihilate each other. However, in numerical applications, the phase space has to be decomposed into cells rather than points, where the annihilation property gives rise to the concept of indistinguishable particles, which are stored in cells at consecutive time steps, so that a single integer number per cell replaces the ensemble of particle states within that cell. This greatly reduces the memory requirements in the implementation of the model. The first two-dimensional Wigner function based simulations could only be realized thanks to this approach but also due to novel parallelization strategies [52,53]. Because of both accomplishments, the number of particles can be significantly increased while still retaining feasible simulation times. 

A variety of applications has been explored using the particle interpretation of the Wigner function formalism. As already discussed, a comparison between NEGF and Wigner simulations of RTD has been presented [54], showing how the Wigner function based approach bridges the gap between purely coherent and scattering dominated transport regimes. In the next section, we give some further illustrative examples of the Wigner Monte Carlo method.

### 4.2. Why Not Non-Equilibrium Green’s Functions (NEGF)?

The NEGF has been widely applied to simulations of semiconductor devices, despite concerns over the applicability of many of the approaches. Some of the more advanced approaches use atomistic approaches to yield the full band structure. This has allowed the use of realistic band structures [55,56,57], phonon spectra [58], strain effects [59], interface roughness [60], and material characteristics. For purely ballistic problems, the NEGF formalism is the approach of choice, as it provides a tomography of the generic physical quantities in terms of energy, although in this situation the problem reduces to merely the retarded and advanced equilibrium Green’s functions [61]. In general, the NEGF approach is efficient for stationary problems—determined by the boundary conditions—near the coherent limit. This is because the center-of-mass coordinates (8) must also be generated in the two-time coordinates. In nearly all work on NEGF, the average position R and the corresponding average time are totally ignored. Hence, these NEGF are for homogeneous steady-state systems. They do not, and often cannot, handle strongly inhomogeneous and transient systems.

There are further problems. Most modern approaches to the use of NEGF base their work on that of Keldysh [62]. The scattering self-energies depend on the carrier $G^{<}$ and $G^{>}$ and on the greater and lesser Green’s functions of the phonons, which account for the occupancy of the phonon states and depend on the phonon energies. While this approach has been widely used, Keldysh points out that it works only upon the assumption that the system is close to equilibrium, so that the normal techniques can be used. For example, Keldysh assumes that the interaction representation, in which the scattering-derived interaction representation is assumed to be a unitary translation operator. But, this assumption fails when the energies contain self-energies so that the Hamiltonian, for example, (2), is no longer Hermitian. Again, most approaches for devices use only the Dyson equation [63] to provide a re-summation of interaction terms into a simple self-energy. With anisotropic scattering, such as that by impurities or polar phonons, one needs to utilize the Bethe–Salpeter equation [64] to completely evaluate the scattering. The onset of nonequilibrium phonon distributions [65], proper treatment of the scattering in many cases, and full use of both the Bethe–Salpeter equation with vertex corrections, increase tremendously the method’s computational effort. It is then easy to see that particle-based Wigner functions allow a quicker route to the solution, as well as being more applicable than NEGF in many cases, particularly when inhomogeneous and transient transport is desired.

## 5. Wigner Function Applications

The Wigner function has been applied to a great many fields of science, so that there are a great many possible examples that could be discussed. Here, we will concentrate on four examples that have been investigated by the authors in a great many studies, and with new results. The first deals with electron tunneling, which has been discussed and treated with Wigner functions for some half a century, but it still admits to new insights. The second example comes from the study of spin transport in semiconductors, in which one common observable is the spin Hall effect, treated here for the first time by Monte Carlo techniques. Our third study deals with Josephson junction circuits, which are of interest to the quantum information and computing world, again treated for the first time by Monte Carlo techniques. Finally, we deal in the fourth example with electron interference in which local potentials, such as those arising from impurities, affect the quantum nature of scattering.

### 5.1. Tunneling

**Figure 2:**
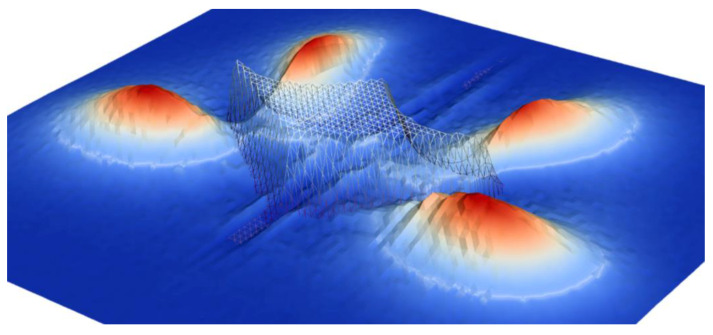
An initial Wigner function corresponding to a minimum uncertainty wave packet encounters a potential with a specifically engineered shape, splitting the initial packet into four well established density peaks propagating in disparate directions. The evolution maintains the initial coherence. The variations of the density in the potential region are related to the oscillations of the Wigner function, which furthermore connote interference effects and entanglement.

In Figure 2, we illustrate just how Wigner functions generate entanglement merely by passing through a tunneling junctions. Here, the simulation is based on the signed-particle model, and can be conveniently understood in classical terms thus enabling a deep physical insight about the evolution process. An initial carrier state encounters a potential with a specifically engineered shape, splitting the initial packet to four well established density peaks. The evolution maintains the initial coherence despite the fact that the peaks propagate in disparate directions. If time is reversed, the backward evolution recovers the initial state. This means that if one of these peaks is modified by another potential, the interaction will be felt by the whole state so that the rest of the peaks are modified too. Any such interaction causes a momentum redistribution of the signed particles representing the carrier state, and that is how we can establish an intuitive picture in which the peaks communicate via the momentum. This picture remains valid as long as the involved physical processes maintain the initial coherence. Phonons strive to redistribute the momentum of the signed particles towards equilibrium [66]. Then, the evolution loses it’s quantum mechanical character in favor of the classical behavior, where an initial carrier ensemble is scattered by the potential, which modifies the probability distribution of the carriers in the phase space. These considerations illustrate processes that are important in, e.g., the area of quantum cryptography, where the primary obstacles are decoherence processes. 

### 5.2. Spin Filtering

Gaps that open in the free electron spectrum in semiconductors typically give band energies of the Einstein (relativistic) form. This is especially true when the spin-orbit interaction is included. In the layer compounds, and particularly in the transition metal di-chalcogenides (TMDCs), in the presence of the spin-orbit interaction, the Hamiltonian can be written as [67]

(14) $$H=at\left(\tau k_{x}\sigma_{x}+k_{y}\sigma_{y}\right)-\frac{\lambda \tau }{2}\left(\sigma_{z}-1\right)s_{z},$$

where the various σ terms are the Pauli matrices for two pseudo-spin basis functions of the valleys in the strange bands of the TMDC, *τ* is the valley pseudo-spin index, *a* is the lattice constant, *t* is the nearest neighbor hopping energy, Δ is the energy gap, 2*λ* is the spin splitting at the valence band top, and *s_z_* is the Pauli matrix for spin. These materials lack an inversion symmetry, and the principal valence and conduction bands around the band gap derive primarily from the transition metal *d*-states [68], and they tend to have a direct band-gap at the *K* and *K’* points (corners of the hexagonal shape) of the Brillouin zone. The spin–orbit interaction produces opposite spin splittings in the two equivalent valleys of the valence band, and can lead to a valley-spin Hall effect, similar to the usual spin Hall effect [69,70,71], arising from the presence of a Berry curvature [72]. The spin is coupled to the valley pseudo-spin due to the difference in the orientation of the spin splitting [73]. This leads to a transverse velocity that is different in the two valleys and pushes the opposite spins to opposite sides of the nanowire.

**Figure 3:**
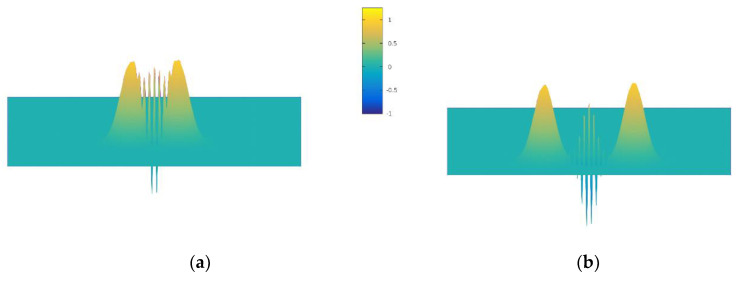
Wigner function for a pair of opposite spin electrons propagating via the valley spin Hall effect. The entanglement is clearly shown between the two main Gaussians. (**a**) shortly after the two spins states separate, where the entanglement still overlaps the main pulses. (**b**) At a later time, when the two main pulses are further separated and the entanglement is more distinct.

In light of the above, it is clear that if we represent the particle by a Gaussian wave packet, it will move with a constant drift velocity that has a longitudinal component due to the applied electric field and a transverse component due to the Berry curvature and effective magnetic field. The latter will be oppositely directed for the two valleys. To illustrate this, we take the TMDC WS_2_. Because the mobility of WS_2_ is only of the order of 10–100 cm^2^/Vs, the transverse velocity is not that much smaller than the longitudinal velocity. For a longitudinal field of 1 kV/cm, a typical density of 10^11^ cm^−2^ of free carriers, a mobility is 60 cm^2^/Vs, we find a drift velocity of about 3.6 × 10^4^ cm/s. The transverse velocity is generated by the Lorentz force arising from the effective magnetic field, and this gives a transverse velocity of approximately 1.9 × 10^4^ cm/s, using values in (15) from [74]. We will use these values below in the simulations. For this system, the Wigner function for two spins starting from the same location in the semiconductor will be entangled with a cross-term representing the correlation of the two oppositely-directed spins [74], and this may be evaluated analytically. Using the particle Monte Carlo approach, we can continue to use a simple model for the Wigner function, formed initially from two Gaussian packets as in the analytical approach. However, we utilize a new sampling technique [75] for the Wigner potential to evaluate the entanglement of the two packets. In this approach, the wave-packet phase-space Monte Carlo method expands the wave-function in a local basis set (e.g., a Gaussian), then acquires via Ehrenfest’s theorem a system of ordinary differential equations similar to the classical Hamilton’s equations. Expectation values are then propagated in a manner similar to classical statistical mechanics. An interference pattern concentrated between the two arises from incorporating a phase related to the differences in real space and momentum space between the two packets. Particles are accelerated by using Ehrenfest’s theorem for a real and quantum potential, whose value is found from the parameters of a parabolic band model of WS_2_, and then utilizing the Hamiltonian Monte Carlo technique for solving the Wigner equation. The results are shown in Figure 3, and agree almost exactly with the analytical results. One may compare these results with those of [74] to observe that the Monte Carlo procedure faithfully reproduces the analytical results. 

### 5.3. Superconducting Josephson Junction

The previous examples are known to be strongly connected to classical phase space solutions. In many cases, the equation simply becomes the Boltzmann equation. The characteristics of the Liouville operator are simply the classical trajectories. Now, we want to turn to non-quadratic Hamiltonians. The only nonlinear Hamiltonian known in quantum mechanics to have an analytical solution is the hydrogen atom. However, it would be of greater interest to see if the method can actually identify the eigenvalues and eigenfunctions for a Hamiltonian with unknown solutions, or at least not well-known solutions. The simplest case to examine is the tilted washboard, given by

(15) $$H\left(x,p\right)=\frac{p^{2}}{2m}-\alpha \cos\left(\beta x\right)-\gamma x.$$

This is often referred to as the cosine-Gordon equation, although it occurs more commonly with a sine term instead of the cosine term. Here, *α* is usually related to the d.c. Josephson current, which exists in the absence of an electric field in the Josephson tunnel junction [76]. The term *β* is related to the magnetic field, in which the Josephson current is oscillatory in the ratio of *BA*/Φ_0_, where *B* is the magnetic field, *A* is the area of the junction, and Φ_0_ (=*h*/2*e*) is the flux quantum (the factor of 2 arises as the tunneling particle is a Cooperon of paired electrons). The term *γ* = *eE* is an applied electric field which gives a tilt to the periodice cosine potential. The cosine, or washboard, potential is commonly used in Josephson based qubits in quantum information.

**Figure 4:**
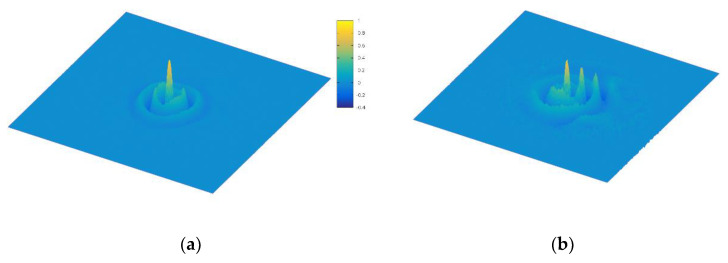
(**a**) Wigner function initialized near E_3_ in the cosine potential, for the cosine-Gordon equation. Here, *γ* = 0.2. (**b**) Wigner function for *γ* = 1.2, illustrating quantum diffusion to adjacent cells of the cosine potential.

To study this, we use the normalized parameters *m* = 1.5625, *β* = 1.0 and *α* = 4.0. We use a numerical eigen solver routine to estimate the first three eigenvalues to be E_0_ = 0.816, E_1_ = 3.25, and E_2_ = 5.5, all in normalized relative units. Instead of sampling the phase space coordinates directly, we instead sample the energy distribution and place the particles near the energy eigenvalue E_3_ of the oscillator to generate the boson Fock state. For low values of *γ*, the system is essentially a bound well and should have eigenstates similar to a Fock state. As *γ* increases, the asymmetry should tilt and shift the eigen spectrum. We illustrate this with two values of *γ*, as shown in Figure 4. In panel (a), we initiate the Wigner function using the *n* = 2 Fock state, then let the particles evolve until a steady state Wigner function is reached. In panel (b), we raise the electric field to induce particles to diffuse into adjacent minima of the cosine potential, thus leading to quantum diffusion. In each case, as the tilt is increased, we see the Wigner function increasingly skewed from the excited harmonic oscillator we initialize the particles with.

It was mentioned above that the tilted washboard potential is a model of the superconducting qubit Hamiltonian. It is straightforward to couple multiple qubits together with a pairwise interaction. Quantum circuits for quantum computing, such as the one recently unveiled by Google, involve only a small number of quantum particles, in their case 53 [77]. Generally, quantum simulation algorithms scale at best O(exp(*N*)), where *N* is the number of particles. For the present algorithm, which is a full solver for the Wigner equation, the scaling is O(*N_p_^3^*), where *N_p_* is the number of pseudo-particles. The number of pseudo-particles is very challenging to estimate. The Wigner function is not a positive definite probability function, so one cannot use typical asymptotic convergence arguments to deduce the Monte Carlo convergence rate. Moreover, the number of particles per quantum particle in the present simulations is at least 10^5^. In the worst case scenario, Google’s quantum circuit would require 5.3 × 10^6^ particles, assuming the qubit-qubit interaction does not necessitate far more particles for convergence. Such a simulation would be tractable on a small number of compute nodes, suggesting that simulating portions of the Google circuit using the above method is a plausible application to explore in the future. 

### 5.4. Magnetic Single-Electron Control in an Interfering Double-Well Potential

Coherent single electron control is critically important for quantum information processing and advanced logic device operation principles based on the quantum character of the electron evolution on the nanometer scale [78]. An interesting and novel mechanism to coherently control single electron dynamics is provided by magnetic double-well potential structures [79]. Here, we investigate the effect of a uniform magnetic field on the electron state interference pattern manifesting in a double-well potential waveguide by means of full Wigner quantum transport simulations.

To be able to clearly investigate the electron quantum dynamics, we focus on coherent transport, i.e., no scattering processes are considered. In extension to previous work, here we target a non-focusing potential well setup, inspired by [80]. We consider the evolution of an initial electron state described by the Wigner function in a two-dimensional phase space ($r=x,y;p=k_{x},k_{y}$) in the presence of a uniform magnetic field B [81] and simulated by the Wigner EMC method using the signed-particle model via ViennaWD [82].

The governing evolution equation is obtained by introducing the magnetic component of the Lorentz force in analogy with the classical (Boltzmann) equation. Indeed, it is not an approximation but an exact quantum-coherent model obtained from the general magnetic Wigner theory for the case of a spatially-dependent but near stationary electric field E(r) and a constant magnetic field B. 

**Figure 5:**
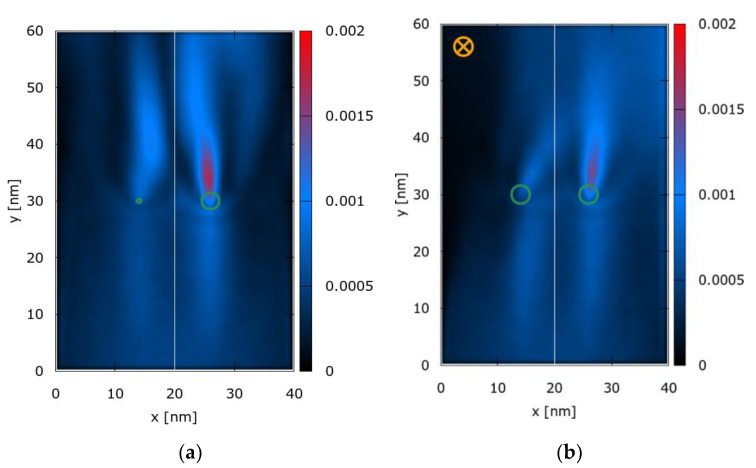
Averaged electron density (arbitrary units): (**a**) Asymetric potential wells and no magnetic field; (**b**) Symmetric potential wells and applied magnetic field. Green isolines indicate potential wells.

Figure 5 shows the principal details of the geometry of the simulated waveguide defined by infinite potentials along the left and right boundary as well as the averaged electron density distribution for symmetrically-sized potential wells and with, and without, a magnetic field. The boundaries in the vertical transport direction (top and bottom) are open. Green isolines at −0.15 eV indicate the potentials wells (Coulomb profile; peak potential at −0.35 eV). The initial state of the electron is the Wigner function corresponding to a minimum uncertainty wave packet with a standard deviation of σ=16 nm . The central wave vector is $\left(k_{0x},k_{0y}\right)=\left(0,0.837\;{\mathrm{nm}}^{-1}\right)$ and corresponds to an energy of 0.14 eV . The initial state is centered at (x=20 nm , y=0 nm) and is injected at the bottom boundary, directed upwards towards the wells. Electrons are injected every femtosecond and do not interact with each other: The electron injections represent independent, identically distributed trials, similar to the Young-type double-slit experiments.

As has been previously shown and as expected, symmetric potential wells give rise to a symmetric electron density interference pattern. However, reducing a potential well by 50% bends the density pattern to the right and shows how the pattern can be manipulated by a potential well induced electric field (Figure 5a). A similar but stronger behavior is observed if the magnetic field is enabled: Figure 5b corresponds to the symmetric double-well potential case with an applied magnetic field (B=−6 T ). The magnetic field shifts the density pattern in a more pronounced way than the asymmetric case. Both effects, the magnetic field and the asymmetric potentials, can be combined to work in tandem to further shift the pattern to the right.

**Figure 6:**
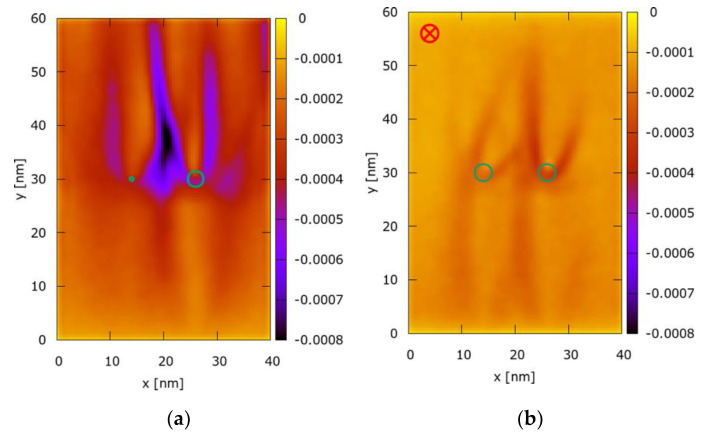
Wigner function negativity map: (**a**) Asymetric potential wells and no magnetic field; (**b**) Symmetric potential wells and applied magnetic field. Green isolines indicate potential wells.

However, despite the similarity of the electric and magnetic effects on the quantum electron density distribution, we observe that the electric and magnetic fields play a very different role in the transport dynamics. Figure 6 illustrates the Wigner function negativity maps corresponding to the setups shown in Figure 5. As previously mentioned, the Wigner function develops negative values in regions of quantum correlation effects. The maps $f_{W}^{-}\left(x,y\right)$ are created by integrating the negative values of the corresponding Wigner functions over the momentum coordinates. As Figure 5 clearly shows, the magnetic field destroys the coherence of the dynamics as the negativity is drastically reduced. This can be linked to the role of the two responsible electromagnetic terms in the transport equation: The action of E is independent from the particle momentum so that the particles in the ensemble are accelerated synchronously. To the contrary, the action of B explicitly depends on the momentum, which distorts the evolution.

## 6. Conclusions

Carrier transport in complex quantum systems, such as nanoscale semiconductor devices embedded within a complicated environment, requires a complex quantum description, which takes into account quantum-coherent phenomena, as well as dissipative processes of scattering, with both modified by the interaction with the environment. The use of the Wigner function is particularly useful in studies of these systems, as the Wigner function may explicitly illustrate the important quantum effects and entanglement that is a signature of quantum interactions. Particle approaches to Monte Carlo simulation of the quantum Wigner function provide an efficient approach to the study of such methods. In these methods, it becomes clear how to study each process and establish its importance in the behavior of the overall system. The Wigner function allows one to clearly identify the quantum effects, particularly the entanglement that arises between different parts of the quantum system.
